# The border-associated macrophage marker MRC1 contributes to an early neuroprotective inflammatory response to traumatic brain injury in mice

**DOI:** 10.1186/s40478-025-02156-z

**Published:** 2025-10-30

**Authors:** Jenny Strehle, Pawit Somnuke, Shuailong Li, Sudena Wang, Tobias Hirnet, Yong Wang, Michael K. E. Schäfer

**Affiliations:** 1 Department of Anesthesiology, University Medical Center, Johannes Gutenberg-University Mainz, Langenbeckstraße 1 (Bld. 505), 55131 Mainz, Germany; 2 Focus Program Translational Neurosciences (FTN), Johannes Gutenberg-University Mainz, Mainz, Germany; 3 Research Center for Immunotherapy (FZI), Johannes Gutenberg-University Mainz, Mainz, Germany

**Keywords:** MRC1, CD206, CNS-associated macrophages, Border-associated macrophages, Traumatic brain injury, Structural brain damage, Neuroinflammation, Microglia, Astrocytes

## Abstract

Macrophages are crucial for neuroinflammatory responses following traumatic brain injury (TBI), encompassing various subtypes, such as border-associated macrophages (BAMs) that contribute to both brain damage and repair. However, the pathophysiological relevance of subtype-specific molecular markers is poorly understood. This study investigated the role of the BAM marker mannose receptor C-type 1 (MRC1, also known as CD206) during the early phase of TBI using controlled cortical impact (CCI). MRC1 gene expression was up-regulated, peaking between 3 to 7 days post-injury (dpi), and MRC1 protein expression predominantly localized to BAMs. To assess pathophysiological relevance, MRC1-deficient (MRC1-KO) and wild-type littermates (MRC1-WT) were examined following CCI for early neurological deficits, brain structural damage, intracerebral hematoma, and neuroinflammatory marker expression. At 5 dpi, MRC1-KO mice showed increased brain lesion volume and hippocampal neuron loss, with minor differences in neurological deficits compared to MRC1-WT mice. Intracerebral hematoma size increased in male but remained unchanged in female MRC1-KO mice. Immunostaining revealed no genotype-specific effects on GFAP^+^ astrocytes, while the number of perilesional CD68^+^ macrophages/microglia were reduced in MRC1-KO mice. Analysis of neuroinflammatory gene markers revealed an overall reduction in MRC1-KO mice. Sex-specific regulation was observed for the M2-like macrophage/microglia marker Arg1, with decreased expression in male and increased expression in female MRC1-KO compared to MRC1-WT mice. In conclusion, lack of MRC1 exacerbated brain tissue damage following experimental TBI. Reduced CD68+ macrophages/microglia and neuroinflammatory marker expression suggests impaired neuroinflammatory response in MRC1-KO, indicating MRC1 expression on BAMs contributes to beneficial early neuroinflammatory response following TBI.

**Supplementary Information:**

The online version contains supplementary material available at 10.1186/s40478-025-02156-z.

## Introduction

Traumatic brain injury (TBI) is a leading cause of death and disability worldwide [1]. Survivors frequently experience long-term neurological impairment, and a high incidence for co-morbidities including neuropsychiatric and dementia disorders has been reported [2–5]. Despite significant advancements in surgical interventions and the neuro-intensive care management of TBI, therapeutic options targeting the pathogenic mechanisms of TBI remain limited [6, 7] and clinical trials testing potential drugs for treating TBI were not or only moderately successful [8–10]. The translation of pharmacological interventions from animal models to human patients TBI research presents numerous challenges beyond the inherent heterogeneity and complexity of human TBI. These challenges include differences in brain size and structure, the white to gray matter ratio between species [11] as well as temporal aspects of pathobiological processes [12]. Human brains have a significantly larger cortical surface area and gyrification compared to rodent models, which influence the manifestation and propagation of injuries. This limitation has been partially addressed through the use of large animal models, such as pigs [13, 14]. Furthermore, the human brain's extended-range connectivity and more advanced neural networks may exhibit different responses to both injury and potential treatments. Nonetheless, mouse models still display several core pathological changes similar to human TBI, making them valuable for investigating cellular and molecular mechanisms [15, 16]. Among these core features are neuronal cell death, changes in cerebral blood flow, oxygenation, mitochondrial metabolism, disruption of the blood–brain barrier (BBB), and progressive brain tissue damage [17]. The rapid onset and sustained activation and accumulation of inflammatory cells is another core feature of TBI, and this process is primarily driven by brain-resident microglia and astrocytes, along with peripheral immune cells that infiltrate the brain. Together, they create a highly inflammatory environment, followed by yet incompletely understood processes to resolve inflammation and enable reparative actions such as scar formation and functional recovery [18, 19].

Macrophages/microglia (M/M) are primary mediators of both beneficial and detrimental aspects of inflammation following TBI, a phenomenon attributed to their polarization into functionally distinct phenotypes. Traditionally, these phenotypes were classified as either pro-inflammatory (M1) or anti-inflammatory (M2) [20]. However, this dualistic classification is not anymore compatible with the wide repertoire of M/M subsets discovered in the recent years [21–24]. Among the subsets which have attracted considerable interest are central nervous system (CNS)-associated macrophages, also referred to as border-associated macrophages (BAMs) [25–27]. BAMs are located at the interface between the CNS and the periphery, occupying locations such as the leptomeninges, perivascular spaces, and the choroid plexus. They potentially function as sentinel cells, monitoring the CNS environment and providing an initial immunological defense [28–32]. Indeed, data from animal models suggest that BAMs are involved in immune surveillance, antigen presentation and drainage, recruitment of peripheral immune cells, brain tissue clearance, and repair, and associate with onset and progression of various CNS diseases including acute brain injury [25, 33]. Despite ongoing work, the heterogeneity and, most notable, the contextual functional diversity of brain macrophages are only just beginning to be explored, as demonstrated by groundbreaking studies utilizing single cell RNA sequencing [34], and multi-omics studies including spatial transcriptomics [35]. Consequently, investigating the heterogeneity of their temporal dynamics, and their specific contributions to TBI progression and recovery is crucial to offer perspectives for therapeutic approaches.

Among the markers of brain tissue-resident macrophages and BAMs is the mannose receptor C-type 1 (MRC1/CD206, here referred to as MRC1), a member of the C-type lectin (CLEC) family [36–38]. MRC1 binds to and internalizes a variety of ligands including those bearing damage-associated or pathogen-associated patterns [39]. MRC1 has been demonstrated to play a role in endocytosis, pinocytosis, and phagocytosis under healthy and pathological conditions in different cell types such as macrophages, immature dendritic cells, and liver sinusoidal endothelial cells [36, 40–42]. Interestingly, a soluble, membrane-cleaved variant of MRC1 has been associated with the pro-inflammatory activation of macrophage [43], and elevated serum levels of soluble MRC1 are correlated with severity of some inflammatory diseases [44–46].

In the present study, we investigated whether MRC1 contributes to early pathophysiological processes following TBI. We subjected adult male and female MRC1 knock-out (KO) mice and their wild-type (WT) littermates to the controlled cortical impact (CCI) model of TBI, performed behavioral monitoring over 5 days after injury, followed by histopathological, and molecular analyses including the quantification of structural brain damage, intracerebral hematoma formation, neuroinflammatory gene and protein expression markers at 5 days post injury (dpi).

## Methods

### Animals and TBI experiments

All animal experiments were carried out in accordance to the ARRIVE guidelines, the institutional guidelines of the Johannes-Gutenberg-University, Mainz, Germany, and were approved by the animal Care and Ethics Committee of the Landesuntersuchungsamt Rheinland-Pfalz (protocol 23 177-07/G19-1-027).

In this study, adult, 8–12 weeks old, male and female mannose receptor c-type 1 knock-out (MRC1-KO) and mannose receptor c-type 1 wild-type (MRC1-WT) mice (BJ.192P2-Mrc1Mnz/J) [47] were used. MRC1-KO and their WT littermates were obtained from heterozygous breeding in the animal facility of the University Medical Center of the Johannes-Gutenberg-University, Mainz, Germany. Genotyping was performed using small ear punches obtained during the marking process, following a standard protocol (Protocol 24,213: Standard PCR Assay Mrc1 < tm1Mnz > alternate1, version 1.2, Jackson Laboratory). All animals were re-genotyped from tail tip material after euthanasia to confirm the genotype. In addition, brain tissue samples from a previous cohort consisting of 50 adult, 10 weeks old, male C57BL6/N mice (protocol 23 177-07/G14-1-037) were used to examine the time-dependent regulation of Mrc1 gene expression and brain distribution of MRC1 + cells [48].

MRC1-WT or MRC1-KO mice were housed and maintained at controlled environmental conditions (22 ± 1 °C, 50 ± 5% humidity, 12 h light/dark cycle) with access to water and food ad libitum. Animals were kept in groups of two per cage. CCI and sham surgeries of MRC1-WT and MRC1-KO mice were performed during daytime essentially as described [49] and analgesic treatment with Carprofen (4mg/kg bodyweight) was applied to all mice regardless of the experimental group at least 30 min prior to surgery. Anesthesia was induced by 4 vol% isoflurane for 90 s and maintained with 1.5–2.1 vol% isoflurane for the whole surgical procedure. After loss of all relevant reflexes, either CCI or sham surgery was performed. Only CCI mice were subjected to Lidocain-hydrochloride subcutaneously to the temples and mice were fixated in a stereotactic frame (Kopf instruments, California, U.S.A.) by shore-bars. Sham animals remained unfixed in prone position during the operation. Bepanthen® eye and nose ointment was applied to eyes and anal region. Body temperature was maintained at 37 °C using a feedback heating plate (Thermostar, RWD life science, San Diego, U.S.A.) during the entire procedure. After surgery all animals were transferred into a temperature (37 °C) controlled incubator (IC8000, Draeger, Luebeck, Germany) and placed back fully awake and orientated to their home cages after two hours. Surgical parameters such as operation time and rectal temperature were recorded before and during surgery and were not different between genotypes (supplementary information, table S1). One male MRC1-KO animal died shortly after CCI and therefore did not reach the planned survival time of 5 days after trauma.

### Experimental design

**Fig. 1:**
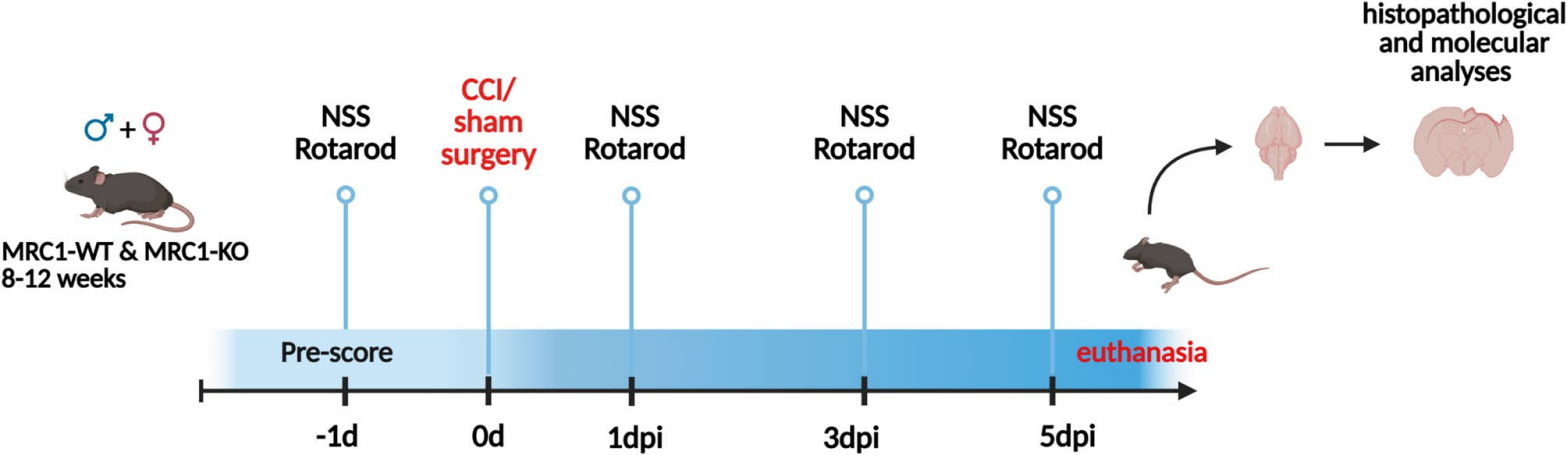
Experimental study design. 5 dpi survival. Neurological Severity Score (NSS) and Rotarod performance (RR) were conducted at 1 day before CCI, at 1, 3 and 5 dpi. At 5 dpi, animals were euthanized, brains were dissected and processed for histological and molecular biological analyses. Created with biorender.com

In total 48 MRC1-WT or -KO mice underwent CCI or sham procedure and were behaviorally assessed over a survival time of 5 dpi followed by histological and molecular analyses (Fig. 1). Mice were randomly divided into four experimental groups with equal sex distribution. The CCI groups (CCI + MRC1-WT and CCI + MRC1-KO) consisted of 16 animals and the sham groups (sham + MRC1-WT and sham + MRC1-KO) consisted of 8 mice. Calculation of animal numbers in CCI groups was based on the assumption that a 20% change in brain lesion size as the main outcome parameter is relevant. The probability of type-1 error was set to α = 0.05, and the probability of type-2 error was set at β = 0.2. One experimenter performed all surgical procedures without knowledge of the genotype, while other experimenters blinded to genotype and surgical procedure assessed the animal’s behavior and performed subsequent data collection and analysis.

### Behavioral assessment

All behavioral experiments that serve to evaluate neurological deficits were performed during daytime in the same order as the CCI/ sham surgeries. To assess neurological functions such as motor skills, coordination, balance and general behavior, a neurological severity score (NSS) was performed one day before surgery (pre-score) and 1, 3 and 5 days post-injury, essentially as described [50]. The minimum score is 0, while the highest possible score is 12. Higher score indicates a more serious injury. The pre-score was performed to exclude neurologically impaired animals and to create baseline for each animal. No animal had to be excluded because of neurological abnormalities.

Rotarod test (RR) was conducted to evaluate motor coordination and balance as previously described [50]. Briefly, mice were placed on an accelerating RotaRod (Panlab RotaRod, Harvard Apparatus, Holliston, MA) and the latency to fall was recorded. Pre-score one day prior to surgery included four training trials, followed by two test trials to quantify the pre-surgery RR performance. After CCI/ sham surgery RR performance was tested at the same time points as NSS and included two runs on the RR and the longest latency to fall out of the two trials was recorded and taken as best performance of the day.

### Brain sectioning and histopathological analyses

Mice were deeply anesthetized with 4 vol% Isoflurane and euthanized by decapitation after an observation period of 5 days. Brains were carefully removed from the skull, frozen in powdered dry ice and stored at -20°C. Using a cryotome (HM 560 Cryo-Star, Thermo Fisher Scientific, Walldorf, Germany) coronal sections were cut and collected as previously described in detail [51]. The collected samples were later processed for RNA extraction and qPCR analyses.

To perform brain volumetry and assess structural damage, cresyl violet staining and hematoxylin–eosin (H&E) staining for hematoma assessment were performed as previously described [51] and the sections were imaged using a microscope equipped with a bright-field camera (Stemi 305, Zeiss, Oberkochen, Germany). Zen software (Zen blue lite, Zeiss, RRID:SCR_013672) was used to quantify lesion volume and granule cell layer (GCL) width and ImageJ software (RRID: SCR_003070) was used to measure the size of the hematoma area [51]. Briefly, brain lesion volumes were determined by identifying areas lacking violet staining or missing tissue in the injured hemisphere from 16 consecutive brain cryosections and subsequently multiplying the intervals between two sections (500 μm). The percentage lesion volume was then calculated by dividing the lesion volume by the total ipsilateral hemisphere volume [52] to avoid possible sex- and genotype-dependent confounding effects due to differences in absolute hemispheric volumes (supplementary information, table S3). GCL width was measured in the dorsal hippocampus at three pre-defined locations in two consecutive sections (~ Bregma level − 1.82 mm to − 2.18 mm) in the suprapyramidal blade of the dentate gyrus of each animal and section. Ipsi- and contralateral GCL were measured and values are expressed in % ipsilesional relative to the contralesional GCL width.

Analysis of the H&E images involved conversion to 8-bit and thresholding using ImageJ. Hematomas were assessed based on the size of the hematoma area. Intensely pink areas were selected using the magic wand. For each animal, four consecutive sections (~ Bregma level + 0.14 mm to -1.36 mm) were measured, and the mean area average was calculated and expressed in mm^2^. Immunofluorescence staining was performed on cryosections, air-dried for one hour, fixed for 10 min in 4% PFA and then incubated in blocking solution, composed of 5% normal goat serum, 0.5% bovine serum and 0.1% Triton X-100 in PBS, for 1h at room temperature. Primary antibodies (Table 1) were diluted and applied in blocking solution over night at 4°C. Next day sections were first washed in PBS, then incubated with secondary antibodies (Table 2) also diluted in blocking solution for 2h at room temperature and afterwards mounted in ImmunoMount (ThermoFisher Scientific, Massachusetts, USA). Immunostainings of brain sections including the fluorophore-conjugated primary antibodies BV421-MRC1/CD206 or BV421-CD68 was performed after incubations with non-fluorophore-conjugated primary antibodies and subsequent fluorophore-conjugated secondary antibody incubations. To control for non-specific antibody binding, fluorophore-conjugated secondary antibodies were applied to brain sections in the absence of the primary antibodies, as well as BV421-conjugated isotype controls for BV421-MRC1/CD206 and BV421-CD68 (both rat IgG2a, κ).

**Table 1:** Primary antibodies for immunofluorescence stainings

Target	Host	Dilution	Manufacturer	RRID	Additional information
CD68 (monoclonal)	Rat	1:300	BD Bioscience	AB_2744447	BV421-labelled
Collagen Type 1 (polyclonal)	Rabbit	1:100	Rockland	AB_2074625	
ER-TR7 (monoclonal)	Rat	1:50	Santa Cruz	AB_1122890	
Iba1 (polyclonal)	Guinea-pig	1:500	Synaptic Systems	AB_2493179	
GFAP (polyclonal)	Rabbit	1:1000	DAKO	AB_10013382	
Ki-67 (D3B5) (monoclonal)	Rabbit	1:500	Cell Signaling Technology	AB_2687446	
MRC1/CD206(monoclonal)	Rat	1:300	BioLegend	AB_10900988	FITC-labelled
MRC1/CD206 (monoclonal)	Rat	1:100	BioLegend	AB_2562232	BV421-labelled
MRC1/CD206 (monoclonal)	Rat	1:500	BioLegend	AB_10900233	
NeuN (polyclonal)	Guinea pig	1:2000	Synaptic systems	AB_2924930	
NeuN (polyclonal)	Rabbit	1:750	Abcam	AB_10711153

**Table 2:** Secondary antibodies for immunofluorescence stainings

Secondary antibody	Host	Reactivity	Dilution	Manufacturer	RRID
Alexa Fluor 633 highly cross-absorbed	goat	guinea pig	1:500	Life Technologies GmbH	AB_2535757
Alexa Fluor 568 cross-absorbed	goat	rabbit	1:500	Life Technologies GmbH	AB_143157
Alexa Fluor 568 cross-adsorbed	goat	rat	1:500	Life Technologies GmbH	AB_2534121
Alexa Fluor 488 highly cross-adsorbed	goat	rabbit	1:500	Life Technologies GmbH	AB_2576217

Images were taken using a fluorescence microscope (BZ-X 800, KEYENCE, Osaka, Japan). Acquisition parameters were individually adjusted to achieve best image quality. The analysis of the triple immunostaining (anti-CD68/anti-GFAP/anti-NeuN) was conducted with ImageJ software (ImageJ, RRID:SCR_003070) and three brain sections (~ Bregma level:  − 1.8 mm to − 2.8 mm) for each animal using adequate threshold setting followed by the “analyze particles”-function. The results were presented either as immunopositive cell number/mm^2^ or as % area of immunopositive cells depending on the analyzed cell type. The analyses of the midline region were conducted from two brain sections (~ Bregma level: + 0.14 mm and − 0.46 mm) and manual counting of anti-MRC1 immunostained cells along the midline from images acquired using a confocal microscope (LSM5 Exciter, Zeiss, 20 × objective). Results were presented as MRC1^+^ cells/mm midline length.

### Gene expression analyses

Brain tissue samples from the right upper quadrant, encompassing the lesion and perilesional tissue, were obtained during histological sectioning. The samples were rapidly frozen in liquid nitrogen, stored at − 80°C, and subsequently processed for RNA extraction, cDNA synthesis using 1 µg RNA, and qRT-PCR as previously described [51] using RNeasy Kit and QuantiTect Reverse Transcription Kits (both Qiagen, Hilden, Germany) in accordance with the manufacturer's instructions. Target specific standard curves of mRNA copies were used to quantify gene expressions including qRT-PCR amplification factor efficiencies (E) calculations for each gene according to the equation *E* = 10^−1/slope^ [53, 54]. SYBR Green and PrimaQuant with matching primers were used for polymerase chain reaction (qPCR) analyses (Light Cycler480, Hoffmann-La Roche AG, RRID: SCR_012155). Samples were analyzed in duplicates using 1µl of cDNA for each target per reaction. First absolute values of gene expression were quantified and then normalized to absolute value of the reference gene *Peptidylprolyl isomerase A* (*Ppia*). Applied primer pair sequences in alphabetically order are provided in Table 3.

**Table 3:** Primer pairs

Gene name, (amplicon size, bp)	Oligonucleotide sequences 5′–3′ (fw: forward, rev: reverse)	Gene bank number
*Aif1 (144)*	fw: ATCAACAAGCAATTCCTCGATGA rev: CAGCATTCGCTTCAAGGACATA	NM_019467
*Arg1* (185)	fw: CTCCAAGCCAAAGTCCTTAGAG rev: AGGAGCTGTCATTAGGGACATC	NM_007482
*Cd68* (113)	fw: CCCACCTGTCTCTCTCATTTC rev: CACATTGTATTCCACCGCC	NM_001291058.1
*Cd74 *[84]	fw: CCGCCTAGACAAGCTGACC rev: ACAGGTTTGGCAGATTTCGGA	NM_001042605
*Fcgr1* (211)	fw: CCACAATGATTGGCTGCTACT rev: CGTGCCTGAGCAGTGGTA	NM_010186.5
*Gfap* (120)	fw: CGGAGACGCATCACCTCTG rev: TGGAGGAGTCATTCGAGACAA	NM_001131020
*Lyz2 (237)*	fw: ACTCCTCCTGCTTTCTGTC rev: TTGCCATCATTACACCAGTATC	NM_017372.3
*Mmp9 (106)*	fw: AAGTCTCAGAAGGTGGAT rev: AATAGGCTTTGTCTTGGTA	NM_013599
*Mrc1* (184)	fw: GGCTGATTACGAGCAGTGGA rev: ATGCCAGGGTCACCTTTCAG	NM_008625.2
*Nos2 (312)*	fw: TGT GTC AGC CCT CAG AGT AC rev: CAC TGA CAC TYC GCA CAA	NM_010927
*Ppia* (146)	fw: gCgTCSCTTCgAgCTgTT rev: RAAgTCACCACCCTggCA	NM_008907
*Spp1 (151)*	fw: ATGTCATCCCTGTTGCCCAG rev: GACTGATCGGCACTCTCCTG	NM_001204201.1

### Statistical analysis

GraphPad Prism software (GraphPad Software Inc., version 9.0) was used to analyze all data. Data distribution was analyzed by Shapiro–Wilk normality test. Outliers were identified by Rout’s test and excluded from further evaluation as indicated in the figure legends. Comparison between two groups were calculated by unpaired student’s t-test for parametric data or Mann–Whitney U test for non-parametric data. Comparative analyses of more than two groups were performed by ordinary one-way analysis of variance (ANOVA) or Kruskal–Wallis test followed by followed by Holm-Šídák or Dunn’s multiple comparison post-hoc test, depending on data distribution. For specific behavioral tests (NSS and RR) and body weight measurements, where more than two groups are evaluated at multiple time points, two-way ANOVA followed by Holm-Šídák post-hoc test, was performed. Values are presented as mean ± standard error of the mean (SEM), *p* < 0.05 was considered as statistically significant.

## Results

### MRC1 gene and protein expression in BAMs is upregulated in the early phase of experimental TBI

**Fig. 2:**
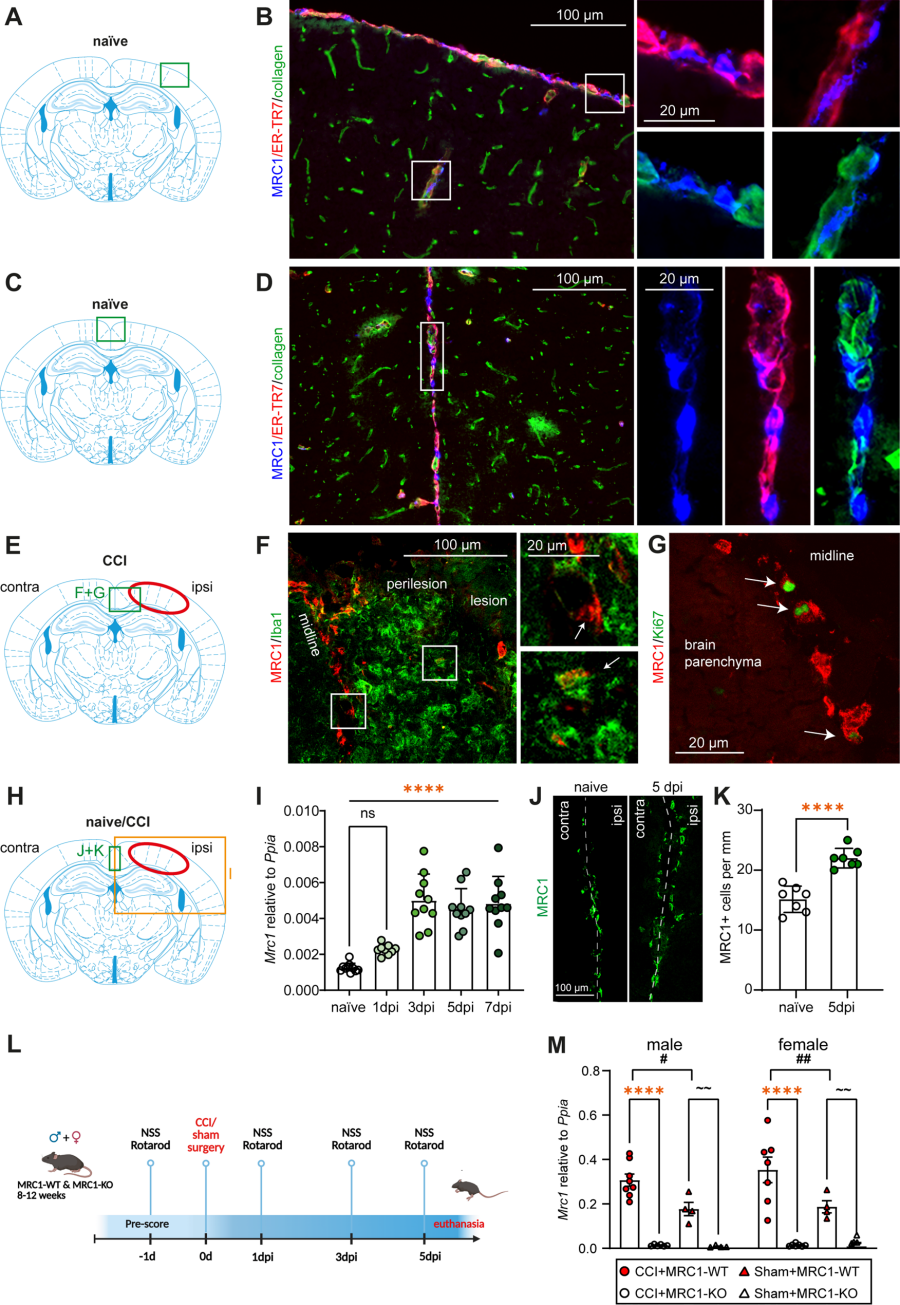
MRC1 gene and protein expression in BAMs is upregulated in the early phase of experimental TBI. Animals were investigated at 5 dpi. **A**, **C** Schematic brain sections illustrating the imaged regions of interest (ROI) shown in **B** and **D**. **B**, **D** Triple-immunostaining of naïve mice with antibodies to MRC1/ER-TR7/collagen in the parietal cortex and midline shows MRC1^+^ cells located adjacent to meningeal fibroblasts (ER-TR7) and large blood vessels (collagen type 1). **E** Schematic brain sections illustrating the imaged ROI shown in **F** and **G**. **F** MRC1 was predominantly expressed by cells aligning the hemispheric midline. Additionally, a small number of vesicle-like MRC1 colocalized with Iba-1 immunopositive cells in the perilesional brain tissue (lower box). **G** Midline-associated MRC1 cells were co-immunolabeled for the cell proliferation marker Ki67. **H** ROI where the material for qPCR was collected (**I**), where images were taken (**J**) and analyses (**K**) were performed. **I** CCI leads to a significant increase in Mrc1 mRNA expression at 3 dpi, 5 dpi, and 7 dpi. One-way ANOVA with Holm Šídák post-hoc test, n = 9–10, values from individual animals and mean ± SEM are shown (****p < 0.0001). **J**, **K** MRC1 + cell number along the midline is increased at 5 dpi compared to naïve mice. Values ± SEM from individual mice are shown (n = 7 per group). Student’s t test, unpaired, two tailed, ****p < 0.0001. **L** Experimental study design of MRC1-WT versus KO 5 dpi survival. **M** CCI leads to an upregulation of Mrc1 expression compared to their corresponding sham group in male and female mice. Rout's test identified one outlier in the female CCI + WT group, which was excluded from subsequent analyses. Two-way ANOVA with Holm Šídák post-hoc test, CCI: n = 7–8, sham: n = 4. Values from individual animals and mean ± SEM are shown. *shows CCI + MRC1-WT versus CCI + MRC1-KO, *p < 0.05, ****p < 0.0001. #shows CCI versus corresponding sham group, #*p* < 0.05, ##*p* < 0.01. ~ shows Sham + MRC1-WT versus Sham + MRC1-KO, ~  ~ *p* < 0.01

Previous studies reported that MRC1 is expressed by BAMs in the naïve brain [55, 56]. To validate these findings, we performed explorative immunofluorescence staining in brain cryosections from naïve C57BL/6N (Fig. 2B, D) and MRC1-WT and -KO (supplementary information, Fig. S2) mice [47]. MRC1 expression in naïve C57BL/6N mice was identified in specific cells located adjacent to meningeal fibroblasts and large blood vessels, which were immunolabelled with the ER-TR7 antibody and anti-collagen type 1 [57]. The immunostaining of naïve MRC1-WT and -KO mice with the markers anti-MRC1 and -Iba1 showed that brain parenchymal microglia expressing Iba1 were devoid of MRC1 immunostaining suggesting that MRC1 is expressed by BAMs rather than microglia in the naïve mouse brain. Brain sections from MRC1-KO mice did not show any specific staining above background levels, as expected (supplementary information, Fig. S2B).

Consistent with the findings in naïve brain, explorative immunostaining for the same markers in C57BL/6N mice following experimental TBI at 5 dpi showed that MRC1 was predominantly expressed by cells aligning the hemispheric midline (Fig. 2F). A rare observation was a vesicle-like MRC1 immunostaining co-localizing with Iba-1 cells at the perilesional brain tissue (Fig. 2F, arrow in the lower box). Furthermore, we observed that midline-associated MRC1 cells were co-immunolabeled for the cell proliferation marker Ki67 (Fig. 2G, arrows). These explorative observations at 5 dpi, suggested that MRC1 is primarily expressed on BAMs, which exhibit proliferative capacity in the early phase of experimental TBI.

To further investigate expression of MRC1, we additionally examined the mRNA expression regulation of Mrc1 following CCI in ipsilesional brain tissue samples from male C57BL/6N mice collected at 1, 3, 5, and 7 dpi. Compared to samples from naïve, non-injured male mice, Mrc1 mRNA expression was increased at all post-traumatic time points and this difference reached a statistically significant level at 3 dpi, 5 dpi and 7 dpi (Fig. 2I). This result demonstrated that CCI increased the mRNA expression of MRC1 at early post-traumatic time points, justifying further investigation into the role of MRC1 in TBI pathogenesis at 5 dpi. Based on these results, we additionally performed anti-MRC1 immunostaining in mice at 5 dpi as well as naïve mice (Fig. 2J). We found that the number of MRC1^+^ cells along the midline was significantly increased at 5 dpi following CCI compared to naïve (Fig. 2K).

To investigate the relevance of MRC1 in TBI pathogenesis, we utilized a MRC1-deficient mouse model and subjected MRC1-WT and MRC1-KO littermates to CCI. Initially, we examined MRC1 gene expression in the different experimental groups at 5 dpi (Fig. 2L). Consistent with the time course results from 1 to 7 dpi, we observed a significantly increased Mrc1 mRNA expression at 5 dpi in MRC1-WT mice as compared to sham. In addition, the Mrc1 upregulation was observed both in female and male mice (Fig. 2M). As expected, Mrc1 mRNA expression was almost undetectable in MRC1-KO mice. In addition, we reconfirmed genotypes using MRC1 immunostaining in brain cryosections (supplementary information, Fig. S2).

### MRC1-deficiency exacerbates structural brain damage at 5 dpi

**Fig. 3:**
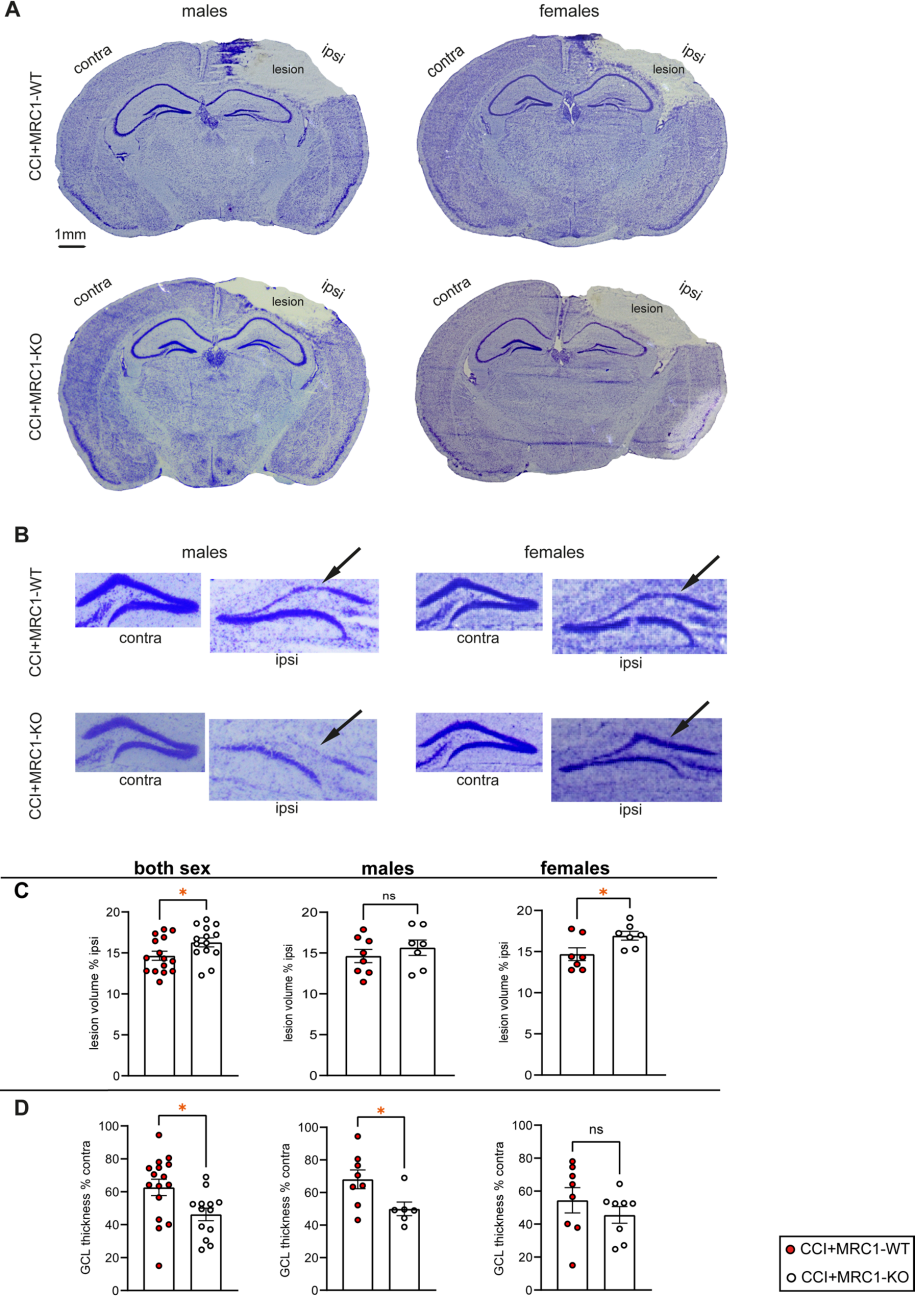
MRC1-KO mice exhibit increased brain tissue loss and hippocampal damage at 5 dpi. **A** Cresyl-violet stained sections from male and female mice showing structural brain damage in MRC1-WT and MRC1-KO CCI groups at 5 dpi. Bregma level: -2.06 mm. **B** Representative image enlargements showing the ipsi- and contralesional GCL of the hippocampus of male and female MRC1-WT and -KO mice. Arrows point to decreased thickness of the ipsilesional GCL. Bregma level: -2.06 mm. **C** Brain tissue loss at 5 dpi. Values (n = 7–16 per group) are expressed in % relative to the volume of the ipsilesional hemisphere. **D** Ipsilesional GCL thickness in MRC1-WT and MRC1-KO CCI groups at 5 dpi (n = 7–16 per group). Values are expressed in % relative to contralesional GCL thickness. **C**, **D** Student’s unpaired t-test or Mann–Whitney U test. Values from individual animals and mean ± SEM are shown. *indicates CCI + MRC1-WT versus CCI + MRC1-KO, *p < 0.05, ns = not significant

CCI produced substantial brain tissue loss at 5 dpi, predominantly affecting the cerebral cortex (Fig. 3A). In addition, an obvious reduction in ipsilesional hippocampal GCL width was observed (Fig. 3B). Determination of brain lesion size by volumetrie in the ipsilesional hemisphere, combining male and female mice, revealed increased brain tissue loss, expressed as percentage volume of the ipsilesional hemisphere, in MRC1-KO mice compared to MRC1-WT mice (Fig. 3C). When data were segregated by sex, similar genotype-specific effects were observed; however, the differences reached the level of statistical significance only in female mice (Fig. 3C). Determination of the relative hippocampal GCL width, combining male and female mice, resulted in a significantly increased loss of GCL in MRC1-KO mice, i.e. decreased ipsi- to contralesional ratio of GCL width (Fig. 3D). This genotype difference also reached the level of statistical significance in male mice, but not in female mice (Fig. 3D). Overall, MRC1-KO mice exhibited an exacerbated structural brain damage at 5 dpi, with deviations observed after sex segregation.

### Lack of MRC1 impairs intracerebral hematoma removal in male mice

**Fig. 4:**
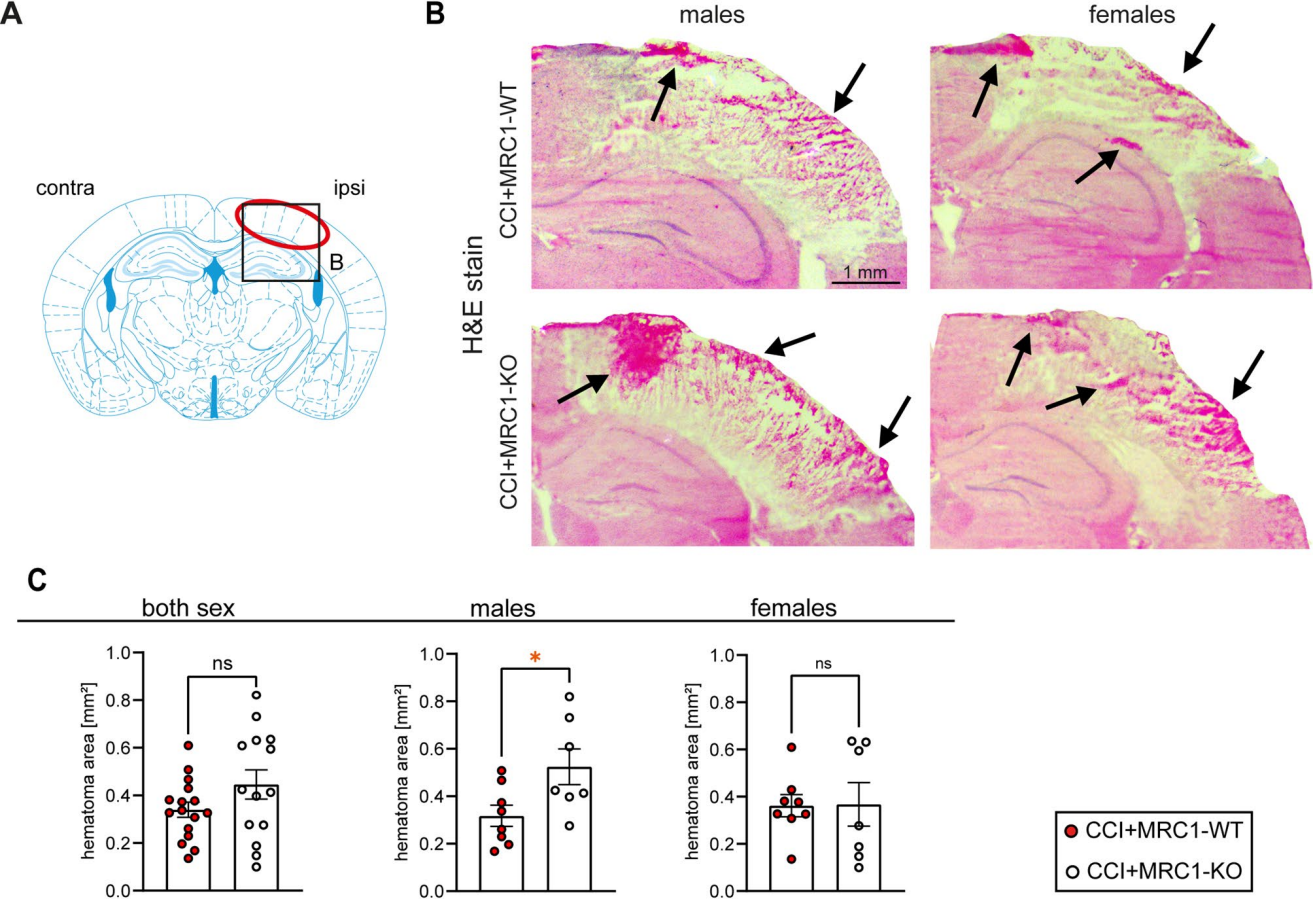
Intracerebral hematoma is more abundant in MRC1-KO mice. **A** Region of interest. **B** H&E-stain shows intracerebral hematoma (arrows) in the ipsilesional brain tissue of CCI + MRC1-WT and CCI + MRC1-WT in male and female mice. Bregma level: -1.36 mm. **C** The hematoma area is increased in CCI + MRC1-KO male mice, but not in female mice as well as both sexes together compared to MRC1-WT group (n = 7–16 per group; four consecutive sections/animal). Student’s unpaired t-test. Values from individual animals and mean ± SEM are shown. *indicates CCI + MRC1-WT versus CCI + MRC1-KO, *p < 0.05, ns = not significant

MRC1 is involved in phagocytosis [58], and our previous study linked phagocytic activity of M/M with the removal of intracerebral hematoma at 5 dpi in the CCI model of TBI [51]. H&E-staining was performed to visualize intracerebral hematomas, which were confined to the cerebral lesion site in both male and female mice at 5 dpi (Fig. 4B). Quantification of the hematoma area in four consecutive sections across the primary impact region, ranging from Bregma + 0.14 mm to -1.36 mm, suggested that male MRC1-KO mice exhibited larger hematoma than male MRC1-WT mice (Fig. 4C). However, this genotype-specific difference was not observed in female mice, suggesting sex-specific differences in the activity of M/M following CCI.

### MRC1 deficiency leads to sex dimorphic regulation of inflammatory gene expression

**Fig. 5:**
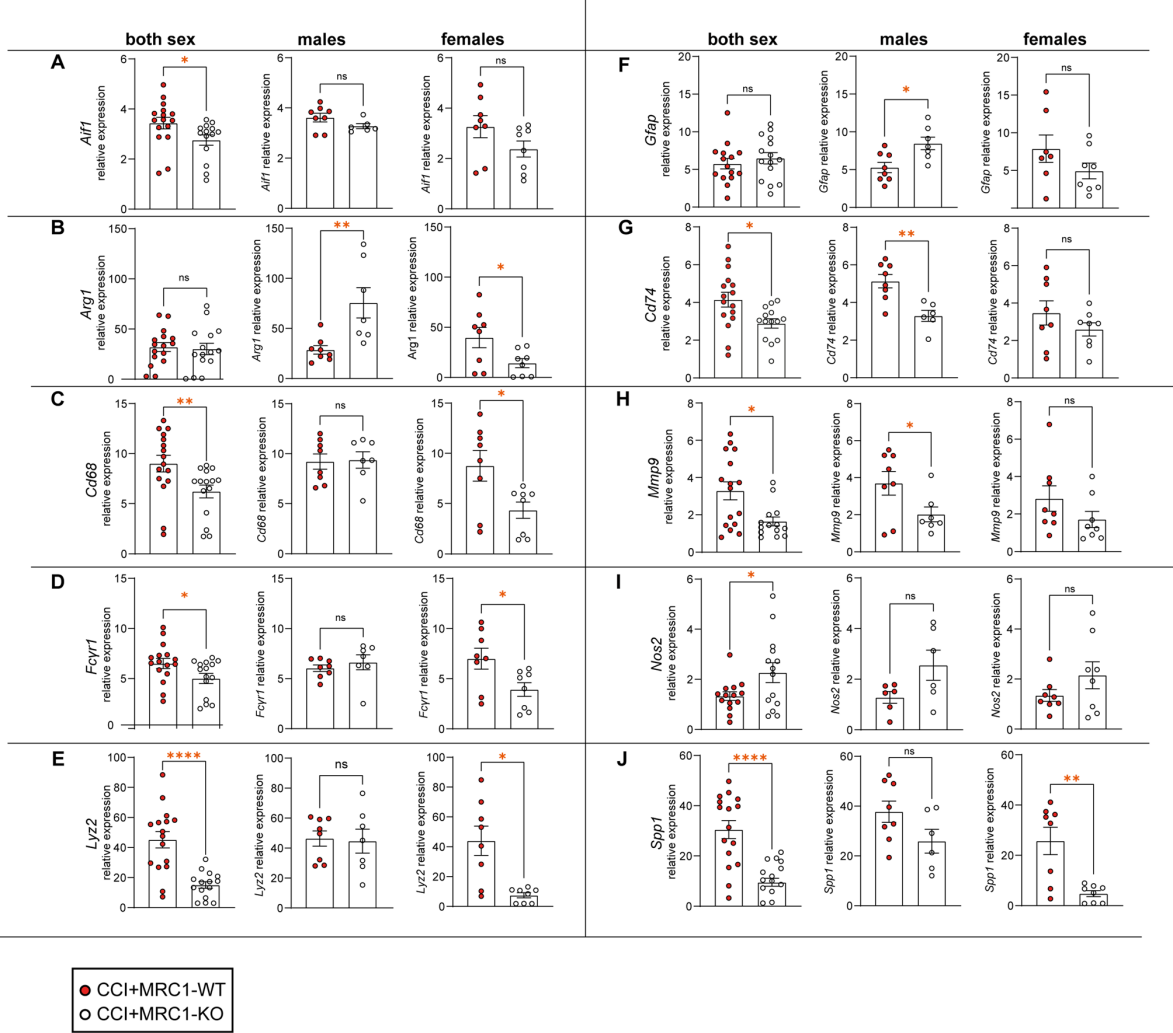
Absence of MRC1 has consequences on the expression of multiple genes and leads to sex-specific differences in the gene expression 5 dpi. **A**–**J** Gene expression analysis in sex-pooled and sex-segregated groups. **A**, **G**, **I**, **J** Rout's test identified one outlier in the sex-pooled CCI + MRC1-KO group, which was excluded from subsequent analyses. **A**–**J** Student’s unpaired t-test or Mann Whitney-U test. CCI: n = 7–8 per group. Values are expressed relative to corresponding sham group. Values from individual animals and mean ± SEM are shown. *indicates CCI + MRC1-WT versus -KO, *p < 0.05, **p < 0.01, ****p < 0.0001, ns = not significant

To investigate consequences of MRC1 deficiency on the neuroinflammatory response to CCI, mRNA expression analyses using qPCR was carried out for inflammatory markers, which we considered to be up-regulated following CCI based on single cell RNA sequencing data deposited in the CEREBRI atlas [24] (https://shiny.crc.pitt.edu/cerebri/). We quantified the relative mRNA expression of markers predominantly associated with M/M, namely, *Aif1*, *Arg1*, *Cd68*, *Fcgr1*, and *Lyz2 *(Fig. 5A–E), the astrocyte activation marker Gfap (Fig. 5F), and markers associated with inflammatory activation in various cell types, namely *Cd74*, *Nos2*, and *Mmp9*, and *Spp1* (Fig. 5G–J). To demonstrate the relative up-regulation in response to CCI, all values were quantified relative to the reference gene *Ppia* and normalized to sham mice of the corresponding genotype and sex. M/M-associated genes were about four- to 30-fold upregulated in response to CCI relative to sham (Fig. 5A–E). Combining values for male and female mice, a significantly reduced mRNA expression was observed for *Aif1, Cd68, Fcgr1 and Lyz2* in MRC1-KO mice compared to MRC1-WT mice (Fig. 5A, C, D, E). However, when segregated by sex, the genotype-specific differences were observed only in female mice, and not in male mice. An opposite regulation was observed for *Arg1* expression. *Arg1* was up-regulated in male MRC1-KO but downregulated in female MRC1-KO mice compared to MRC1-WT (Fig. 5B). A similar trend of opposing regulation was observed for the astrocyte marker gene *Gfap* (Fig. 5F). Genotype-specific differences were also identified for *Cd74*, *Mmp9*, *Nos2*, and *Spp1*, which are gene markers associated with inflammatory activation in various cell types (Fig. 5G–J). While the expression values of *Cd74, Mmp9* and *Spp1* across sexes were reduced in MRC1-KO mice, *Nos2* expression was elevated compared to MRC1-WT (Fig. 5G–J). Sex-segregation exhibited similar regulatory directions for these genes.

Although some sex-specific variations exist in the statistical significance or the direction of gene expression regulation, the overall results of gene expression analysis of various inflammatory marker genes suggest that MRC1 deficiency attenuated the immune response to CCI.

### Lack of MRC1 leads to minor behavioral impairments after CCI

**Fig. 6:**
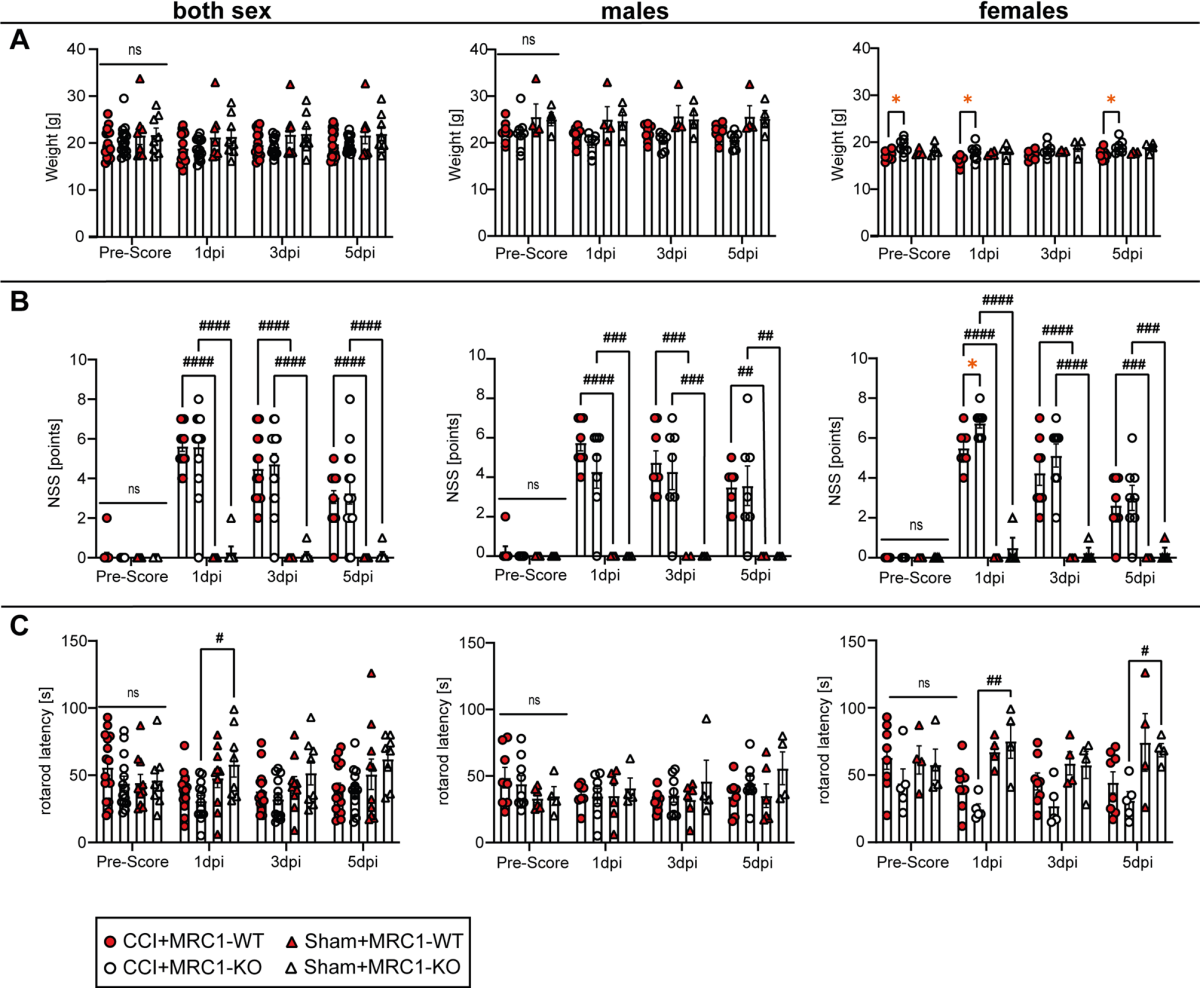
Lack of MRC1 leads to minor behavioral impairments after CCI. **A** Weight development, **B** NSS (higher score indicates increased severity) and **C** RR latency (higher latency indicates better performance). CCI: n = 15–16 per group, sham: n = 8 per group. **A**–**C** Two-Way ANOVA followed by Holm Šídák ‘s multiple comparisons test. Values from individual animals and mean ± SEM are shown. *indicates CCI + MRC1-WT versus CCI + MRC1-KO, *p < 0.05, ns = not significant. ^#^indicates CCI versus corresponding sham group, ^#^*p* < 0.05, ^##^*p* < 0.01, ^###^*p* < 0.001, ^####^*p* < 0.0001, ns = not significant

Body weight evaluation was performed as an indicator of well-being of mice including efficiency of analgesic treatment [59] one day before surgery, at 1 dpi, 3 dpi, and 5 dpi (Fig. 6A and supplementary information, table S2). No significant differences were observed across sexes and CCI or sham groups at any time point (Fig. 6A). However, female MRC1-KO mice randomized to the CCI group exhibited an increased body weight before CCI as well as over the 5 days observation period (Fig. 6A). The effects of CCI and genotype on neurological function were assessed using a Neurological Severity Score (NSS). Sham mice, as expected, showed almost zero points throughout the observation period, regardless of genotype. The NSS reached a peak 1 dpi and subsequently declined until 5 dpi in all CCI groups (Fig. 6B). No genotype-dependent differences were observed across sexes. Female MRC1-KO mice, contrary to male MRC1-KO mice, showed an increased NSS at 1 dpi compared to MRC1-WT; however, this effect was not sustained to 3 dpi or 5 dpi. The RR performance test revealed mild effects on the motor-coordination following CCI, reaching a statistically significant level between CCI versus sham treated MRC1-KO mice across sexes at 1 dpi, in female mice at 1 dpi and 5 dpi (Fig. 6C). Overall, the results suggest that CCI induced more pronounced effects in female MRC1-KO mice than in other CCI groups.

### MRC1 deficiency decreases the number of perilesional macrophages/microglia in the early phase of TBI

**Fig. 7:**
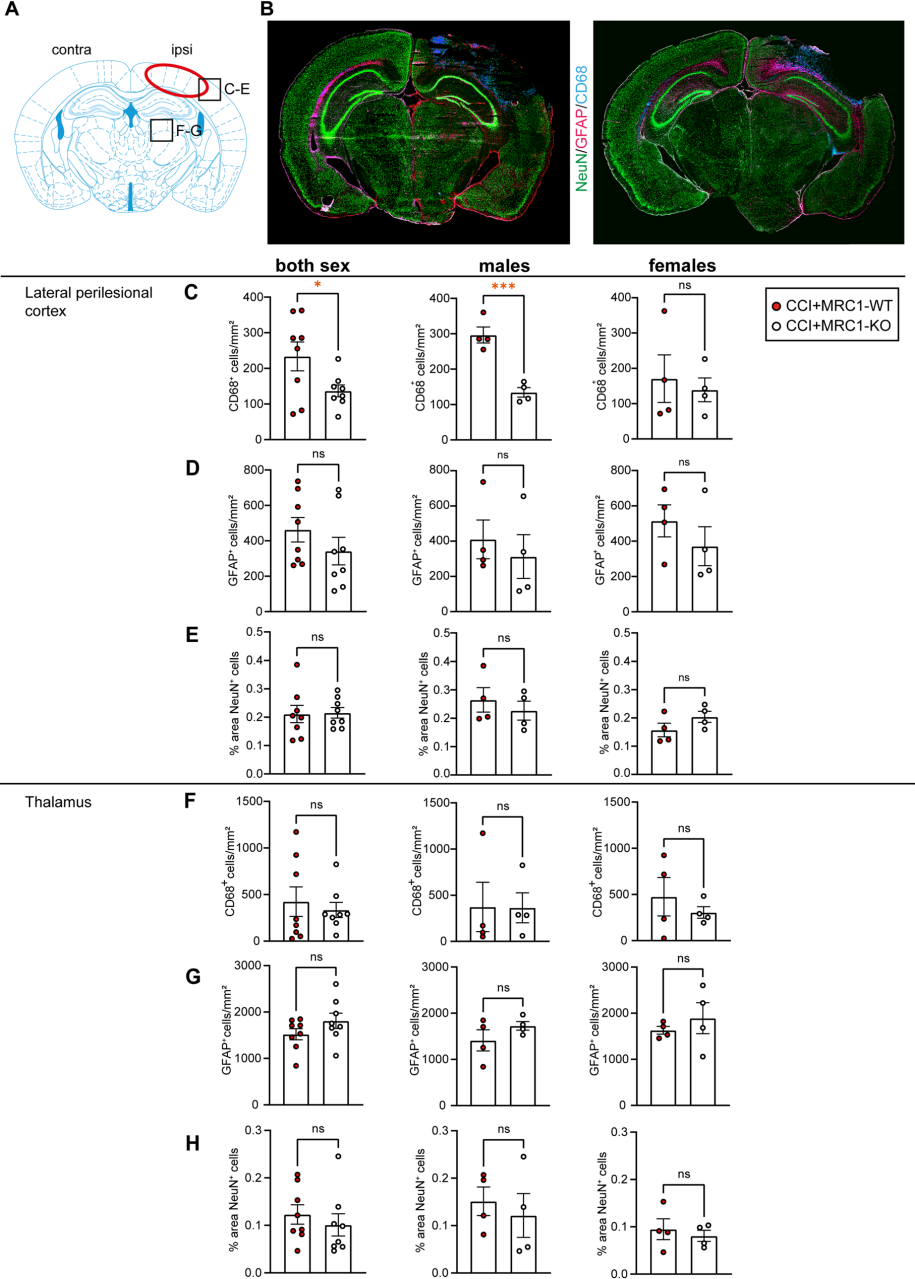
MRC1 deficiency decreases the number of perilesional M/M in the early phase of TBI. **A** Regions of interest. **B** anti-CD68/anti-GFAP/anti-NeuN whole brain immunostaining of CCI + MRC1-WT and CCI + MRC1-KO mice. Bregma level: -2.46mm. **C** Histograms show significantly less CD68 immunopositive cells in the lateral perilesional Cortex of sex-pooled as well as male CCI + MRC1-KO mice. **D**, **E** No differences in the number of GFAP^+^ cells or % area of NeuN^+^ cells in the same brain region. **F**–**H** In the thalamus, however, there were no significant differences between the two genotypes for any of the three markers. CCI: n = 8–16 per group. Student’s unpaired t-test or Mann–Whitney-U test. Values from individual animals and mean ± SEM are shown. *indicates CCI + MRC1-WT versus CCI + MRC1-KO, *p < 0.05, ***p < 0.001, ns = not significant

Activation of glial cells and their recruitment to sites of tissue damage is a hallmark of TBI [19]. Triple-immunofluorescence staining using specific antibodies to CD68, GFAP, or NeuN was performed to investigate possible differences between genotypes in populations of M/M, astrocytes, or neurons (Fig. 7B). As regions of interest (ROIs), we chose the perilesional cortex, primarily affected by CCI, and the dorsal part of the thalamus, which is distant from the primary injury site but infiltrated by M/M [51] (Fig. 7A). In the perilesional cortex, the number of CD68^+^ M/M was significantly reduced in MRC1-KO mice across sexes, and this effect appeared to be largely due to differences in male mice as female mice did not exhibit genotype-specific differences (Fig. 7C). In the thalamus, however, the number of CD68^+^ M/M was not affected by the knock-out (Fig. 7F). No significant differences were observed between genotypes for the number of astrocytes and neurons, neither in the perilesional cortex nor in the dorsal thalamus in our model of TBI (Fig. 7D, E, G, H).

## Discussion

In this study, we investigated the pathophysiological role of MRC1 in the CCI model of TBI. We found upregulated MRC1 gene expression in response to CCI, and immunostaining showed that MRC1 protein expression was restricted to cells at perivascular sites and border regions of the CNS, consistent with previous studies that have identified MRC1 as a marker of perivascular macrophages or BAMs, also referred to as CAMs [25, 31, 56, 60]. Indeed, co-immunostaining using the fibroblast marker anti-ER-TR7 and anti-collagen type 1 [57] revealed that MRC1 + cells were localized to the pial surface and perivascular sites of large blood vessels, which supports their meningeal and extraparenchymal localization. Nonetheless, the application of immunoelectron microscopy would be required to accurately delineate the spatial distribution of MRC1 + cells and their spatial relationship with meningeal and blood vessel-associated cells and anatomical structures such as the Robin-Virchow space [61].

Our results are not entirely consistent with previous observations showing the accumulation of BAMs in brain tissue lesions following TBI in rats [62, 63]. Species-specific differences may account for this observation [64]. In this context, it will be important to determine whether the population of MRC1 + cells observed in this study is attributed solely to BAMs or if it also includes infiltrating cells that express MRC1 upon entering the brain. A bone marrow chimeric MRC1 mouse model would be appropriate for this investigation. Indeed, it was demonstrated that BAMs populations in the brain comprise both locally self-renewed cells and recruited cells from the bone marrow under pathological conditions [65–67].

It should be also noted that detection of MRC1 protein expression on BAMs may be compromised due to proteolytic ectodomain shedding of MRC1, which can be executed by metalloproteases (MMPs), as suggested by pharmacologic inhibition in vitro [68]. In support of this, several MMPs, such as MMP2 and MMP9, have been shown to be upregulated following experimental TBI [69, 70]. Further investigations using fluorescent mRNA labelling technology, antibodies specific for the intracellular part of MRC1, and/or MRC1 transgenic fluorescent reporter mice, for example, would be necessary to address this question in more detail.

Our histopathological analysis demonstrated exacerbated structural brain damage in MRC1-KO mice compared to MRC1-WT mice, whereas gene expression analysis and immunostainings indicated a compromised neuroinflammatory response at 5 dpi. A possible explanation for these observations is a pro-inflammatory function of MRC1. Indeed, MRC1 was shown to induce pro-inflammatory tissue macrophages [43]. Consequently, our results provide evidence that MRC1 contributes to beneficial effects of the early neuroinflammatory response to TBI, which adds to the long-standing research findings regarding the dual nature of neuroinflammation, and particularly M/M, in TBI [71–73]. Moreover, the restricted expression of MRC1 on BAMs suggest their involvement in the early neuroinflammatory response following TBI, which is consistent with the prevailing view that BAMs contribute to the onset of various CNS diseases including acute brain injury [25].

Along this line, several studies linked beneficial effects of neuroinflammation with the clearance of cellular debris including hematoma by phagocytic macrophages/microglial cells [51, 74–78]. Our results showing larger intracerebral hematoma at 5 dpi in male MRC1-KO than in MRC1-WT mice suggest that lack of MRC1 compromises brain tissue clearance. This is consistent with earlier studies identifying MRC1 as a pattern recognition receptor carrying out (macro-) pinocytosis and phagocytosis [41, 79]. Furthermore, inefficient phagocytosis has been considered as a consequence of MRC1 deficiency in other disease models [80]. Notably, this effect was observed exclusively in male mice, indicating that MRC1 may play a role in sex-specific differences in cell tissue clearance and phagocytosis. While recent studies have provided evidence for sex-specific differences in microglia phagocytosis [81, 82], the specific role of MRC1 in this context remains unclear and needs to be investigated in future studies. However, our observations on hematoma size should be interpreted with caution, as hematoma are not uniformly distributed across the injured brain, and we have examined hematoma size in a limited number of sections across the primary impact region. A more comprehensive investigation, utilizing serial sections with minimal intersectional distances followed by stereological analysis, or analysis of optical sections obtained by T2-weighted MRI, is required to examine this question in greater depth.

Moreover, as mentioned, immunofluorescence staining revealed a relatively small number of MRC1^+^ cells, with a restrictive localization that is typical for BAMs [31, 56, 60]. Therefore, MRC1-expressing BAMs may not only directly be involved in the clearance of brain tissue at restricted sites of the brain, but may also play a role in the propagation of inflammatory signals to microglia, thereby facilitating their function in the clearance of cellular debris including hematoma by phagocytosis [51, 74–78]. This is plausible as it has been already established that co-expression of MRC1 with other receptors on macrophages, such as toll-like receptors, can initiate cytokine secretion, e.g. interleukins, subsequently stimulating other immune cells [83].

Further investigations are needed to determine whether, and to what extent, functions of BAMs are modulated by MRC1. For example, employing cell type-specific MRC1 knockout and single-cell RNA sequencing could elucidate cell-autonomous signaling pathways triggered by MRC1, alongside with mouse models specifically targeting BAMs [84]. More insights from such studies on the beneficial effects of MRC1-expressing BAMs during the early phase of TBI may have implications for the development of novel therapeutic strategies for TBI treatment. However, limitations of rodent models related to brain size and structure, including a reduced white to gray matter ratio [11], should be considered.

Moreover, it is of paramount importance to investigate the long-term consequences of MRC1-deficiency on TBI pathogenesis in future studies, as chronic neuroinflammation, including activation of humoral immune responses are critically involved in the patient`s outcome [85, 86]. Furthermore, investigations on the effects of MRC1 deficiency in combination with CCI on other organs are necessary, as peripheral effects of MRC1 deficiency have been reported in other disease models [87, 88].

Our study design, which included both male and female mice, enabled us to explore sex-specific effects of MRC1 deficiency in response to experimental traumatic brain injury. It is worth noting that several studies have also examined sex-specific effects associated with TBI and found evidence of sexual dimorphism in the inflammatory response to TBI. For instance, Villapol et al. demonstrated sex-specific differences after TBI, with male mice exhibiting larger lesion volume than female mice at 3 dpi and 7 dpi and also showed a higher number of infiltrating peripheral myeloid cells in the injured cortex 1 dpi and 3 dpi [89]. Several explanations may account for the less pronounced sex-specific differences observed in our study, including utilization of a different mouse strain, which independently can result in variations in the response to experimental TBI [90]. It is important to note that our statistical study design prioritized genotype-specific differences over sex differences, and a larger sample size may have been necessary to adequately investigate sex-specific differences. Using sex-pooled and -separated analyses, we nevertheless identified genotype-specific effects in a sex-specific manner. These differences were evident in behavior, lesion parameters, gene expression and immunofluorescence staining. As already known, sex steroid hormones, including estrogens, androgens and progestins, are involved in the regulation of overall immune function [91]. Previous studies already showed that this applies not only to general immune function but also to the neuroinflammatory response to TBI. Male mice presented a significantly higher influx of peripheral myeloid cells in the first days after injury, which was followed by proliferation of resident microglia compared to female mice [82]. Additionally, pharmacological inhibition of E2 and DHT synthesis or ovariectomy showed effects on glial activation, other pathobiological processes and behavioral performance in the early phase of TBI [92, 93]. In addition to sex steroids, other factors also influence the neuroinflammatory response in a sex-specific manner. It was reported that microglia are more sensitive to inflammatory stimulation and adopt an inflammatory phenotype in male mice compared to females, independent of sex-steroids [94]. Interestingly, MRC1 expression in M/M appeared to exhibit sex-specific differences in an experimental spinal cord injury model [95] and our gene expression analysis suggested that CCI caused a relatively stronger increase of Mrc1 expression in females than in males.

In conclusion, this study provided evidence that MRC1 expression on BAMs contributes to an early neuroprotective inflammatory response to TBI in mice. This underlines a critical role of BAMs in TBI pathogenesis.

## Supplementary Information

Below is the link to the electronic supplementary material.

Supplementary Material 1

## Data Availability

Source data are available upon reasonable request.
