# Supplementary material for: The border-associated macrophage marker MRC1 contributes to an early neuroprotective inflammatory response to traumatic brain injury in mice
Source: Acta Neuropathol Commun. 2025 Oct 30;13:220. doi: 10.1186/s40478-025-02156-z (PMC12574200; doi:10.1186/s40478-025-02156-z)
Supplement: Supplementary file 1 — Supplementary Material 1 [file 40478_2025_2156_MOESM1_ESM.pdf]

Supplementary data

Table S1: Intraoperative monitoring of CCI and sham mice

	**CCI**						**Sham**					
	MRC1-WT			MRC1-KO			MRC1-WT			MRC1-KO		
	m+f	m	f	m+f	m	f	m+f	m	f	m+f	m	f
**Rectal temperature preoperative [°C]**	36.19 ± 0.14	36.09 ± 0.20	36.28 ± 0.19	36.30 ± 0.14	36.33 ± 0.25	36.28 ± 0.15	36.44 ± 0.28	36.08 ± 0.50	36.8 ± 0.20	36.38 ± 0.21	36.45 ± 0.42	36.30 ± 0.17
**Rectal temperature intraoperative [°C]**	36.48 ± 0.12	36.41 ± 0.22	36.53 ± 0.15	36.51 ± 0.19	36.24 ± 0.34	36.71 ± 0.20	36.24 ± 0.17	36.48 ± 0.14	36.00 ± 0.29	36.33 ± 0.18	36.45 ± 0.32	36.10 ± 0.20
**Duration of operation [min]**	17.06 ± 0.73	17.71 ± 1.19	16.56 ± 0.93	17.75 ± 0.39	17.43 ± 0.48	18.25 ± 0.62	15.63 ± 0.32	15.75 ± 0.48	15.50 ± 0.50	17.88 ± 0.88	17.00 ± 1.23	18.75 ± 1.25

Body temperature was controlled by a feedback heating device adjusted to 37°C intraoperatively. Physiological and technical parameters were monitored before and/or during the surgical procedure. Values are expressed as mean ± SEM. One-way ANOVA followed by Holm Šídák‘s multiple comparisons test or Kruskal-Wallis test followed by Dunns multiple comparison test, depending on the Gaussian distribution, revealed no statistical differences between all groups. M+F = both sex, m = male, f = female.

Table S2: Body weight of CCI and sham mice

(body weight in grams)

	**CCI**						**Sham**					
	MRC1-WT			MRC1-KO			MRC1-WT			MRC1-KO		
	m+f	m	f	m+f	m	f	m+f	m	f	m+f	m	f
**Pre-op**	19.79 ± 0.77	22.31 ± 0.79	17.31 ±0.41	20.60 ± 0.78	22.04 ± 1.31	19.16 ±0.55	21.68 ± 1.95	25.58 ± 2.72	17.78 ±0.35	21.78 ± 1.45	24.80 ± 1.97	18.48 ±0.69
**1 dpi**	18.88 ± 0.80	21.66 ± 0.65	16.09 ± 0.34	18.75 ± 0.56	19.86 ± 0.85	17.79 ± 0.59	21.28 ± 1.90	25.00 ± 2.75	17.55 ± 0.24	21.43 ± 1.55	24.73 ± 1.84	18.13 ± 0.75
**3 dpi**	19.68 ± 0.72	22.11 ± 0.64	17.25 ± 0.35	19.27 ± 0.49	20.37 ± 0.69	18.31 ± 0.50	21.85 ± 1.80	25.73 ± 2.26	17.98 ± 0.12	22.01 ± 1.48	25.10 ± 1.77	18.93 ± 0.87
**5 dpi**	19.85 ± 0.74	22.36 ± 0.64	17.34 ± 0.39	19.79 ± 0.44	20.67 ± 0.66	19.01 ± 0.47	21.68 ± 1.84	25.65 ± 2.29	17.70 ± 0.15	22.01 ± 1.46	25.20 ± 1.71	18.83 ± 0.54

Body weight was measured at pre-determined time points throughout the experiment. Values are expressed as mean ± SEM. Two-way ANOVA followed by Holm Šídák's multiple comparisons test revealed significant differences between female CCI+MRC1-WT and –KO mice pre-op (p = 0.0154), 1dpi (p = 0.0396) and 5dpi (p = 0,0443). M+F = both sex, m = male, f = female.

Table S3: Spatial distribution of brain lesion at 5 dpi (percentage of animals showing lesion)

% of animals with lesion	0	0	7	16.7	7.33	90	100	100	100	100	100	100	96	83	50	16.7
Section	1	2	3	4	5	6	7	8	9	10	11	12	13	14	15	16
Bregma level according to mouse brain atlas (approx.)	~ 3.2 mm	~ 2.7 mm	~ 2.2 mm	~ 1.7 mm	~ 1.2 mm	~ 0.7 mm	~ 0.2 mm	~ -0.3 mm	~ -0.8 mm	~ -1.3 mm	~ -1.8 mm	~ -2.3 mm	~ -2.8 mm	~ -3.3 mm	~ -3.8 mm	~ - 4.3 mm

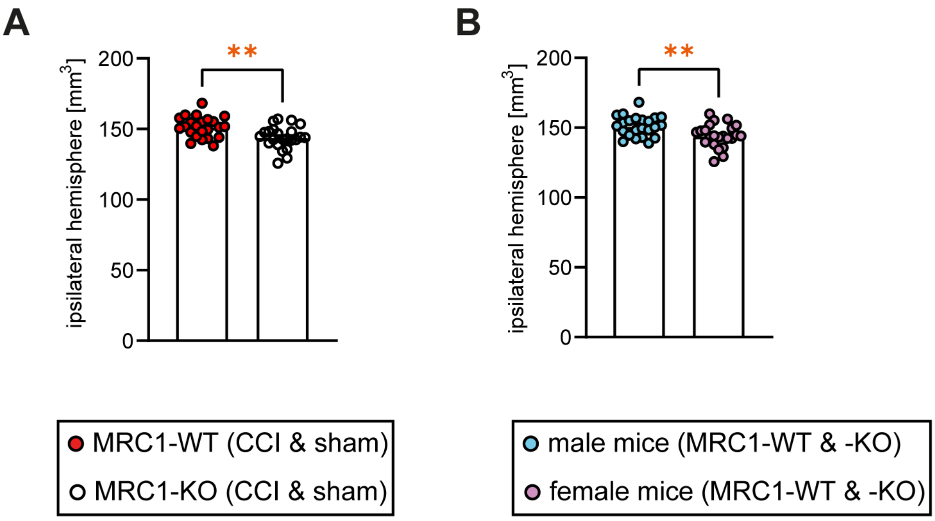

Figure S1: Comparison of the ipsilateral brain volume revealed significant difference between MRC1-WT and –KO mice (A), as well as between male and female mice (B). (A,B) Student's unpaired t-test or Mann Whitney-U test. n = 23 per group. Values from individual animals and mean ± SEM are shown. *indicates CCI+MRC1-WT vs. CCI+MRC1-KO, **p < 0.01.

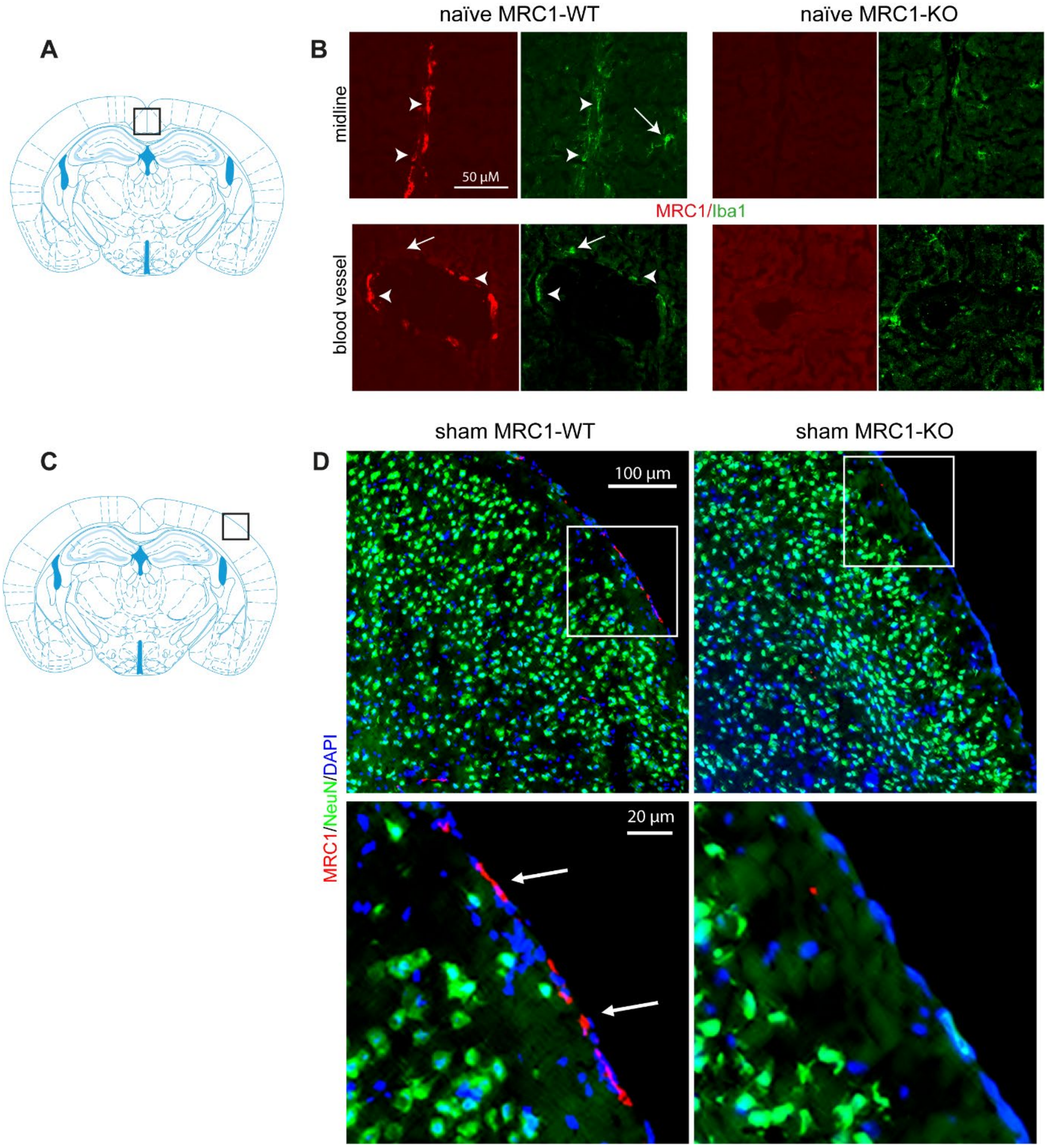

Figure S2: (A, B) Immunostaining of brain cryosections showing the midline region and blood vessels from naïve MRC1-WT and –KO mice. MRC1^{+} cells co-expressed the macrophage/microglia marker Iba1 at these sites. However, brain parenchymal microglia expressing Iba1 were devoid of MRC1 immunostaining suggesting that MRC1 is expressed by BAMs rather than microglia in the naive mouse brain. (C, D) Brain sections from MRC1-WT and MRC1-KO mice (parietal cortex is shown) immunostained with anti-MRC1 and anti-NeuN (counterstained by DAPI) did not show any specific

anti-MRC1 staining in MRC1-KO mice, as expected. Arrows depict MRC1+ cells in the outermost region of the parietal cortex, which were not detectable in MRC1-KO mice. The images in the lower row are an enlargement of the area in the white square of the images in the upper row.
